# Human papillomavirus, vaginal microbiota and metagenomics: the interplay between development and progression of cervical cancer

**DOI:** 10.3389/fmicb.2024.1515258

**Published:** 2025-01-22

**Authors:** Paul Leon-Gomez, Vanessa I. Romero

**Affiliations:** ^1^College of Biological and Environmental Sciences, Universidad San Francisco de Quito, Quito, Ecuador; ^2^School of Medicine, Universidad San Francisco de Quito, Quito, Ecuador

**Keywords:** humanpapillomavirus (HPV), vaginal microbiota, cervical cancer, metagenomics, dysbiosis

## Abstract

Persistent infection with oncogenic human papillomavirus (HPV) types, such as HPV 16 or 18, is a major factor in cervical cancer development. However, only a small percentage of infected women develop cancer, indicating that other factors are involved. Emerging evidence links vaginal microbiota with HPV persistence and cancer progression. Alterations in microbial composition, function, and metabolic pathways may contribute to this process. Despite the potential of metagenomics to explore these interactions, studies on the vaginal microbiota’s role in cervical cancer are limited. This review systematically examines the relationship between cervical microbiota, HPV, and cervical cancer by analyzing studies from PubMed, EBSCO, and Scopus. We highlight how microbial diversity influences HPV persistence and cancer progression, noting that healthy women typically have lower microbiota diversity and higher *Lactobacillus* abundance compared to HPV-infected women, who exhibit increased *Gardenella, Prevotella, Sneathia, Megasphaera, Streptococcus, and Fusobacterium* spp., associated with dysbiosis. We discuss how microbial diversity is associated with HPV persistence and cancer progression, noting that studies suggest healthy women typically have lower microbiota diversity and higher *Lactobacillus* abundance, while HPV-infected women exhibit increased *Gardnerella*, *Prevotella*, *Sneathia*, *Megasphaera*, *Streptococcus*, and *Fusobacterium* spp., indicative of dysbiosis. Potential markers such as *Gardnerella* and *Prevotella* have been identified as potential microbiome biomarkers associated with HPV infection and cervical cancer progression. The review also discusses microbiome-related gene expression changes in cervical cancer patients. However, further research is needed to validate these findings and explore additional microbiome alterations in cancer progression.

## Introduction

Human papillomavirus (HPV) is one of the most common sexually transmitted infections worldwide, particularly in women under 25 (Sharifian et al., 2023). Nearly 90% of women are exposed to HPV during their lifetime, but most infections resolve before viral integration into the host genome (Karpinets et al., 2022; Szymonowicz and Chen, 2020; Karpinets et al., 2020). However, about 10% persist, significantly increasing the risk of cervical cancer (Shulzhenko et al., 2014; Chang et al., 2023; Woodman et al., 2007). High-risk types like HPV 16 and 18 are the leading cause of cervical cancer and are associated with other cancers, such as those of the head and neck (Ure et al., 2022; Koshiol et al., 2008; Castellsagué, 2008; Banerjee et al., 2015; Chen et al., 2020; Condic et al., 2023; Arroyo Mühr et al., 2015; Feng et al., 2008; Zheng et al., 2023).

A dysbiotic cervicovaginal microbiome is more permissive to persistent HPV infection, facilitating viral oncogene expression and subsequent cervical dysplasia and cancer (Fang et al., 2022; Gilbert et al., 2018; Irfan et al., 2020). This dysbiosis is especially relevant among Hispanic women, whose microbiota is often low in *Lactobacillus* and resembles that of HPV-infected women, increasing their vulnerability to persistent infections (Tosado-Rodríguez et al., 2024; Vargas-Robles et al., 2023; Thyagarajan et al., 2020). A “nonoptimal” microbiota, characterized by reduced *Lactobacillus* species and overrepresented anaerobic bacteria and fungi, predisposes this group to cervical dysplasia and malignancy (Godoy-Vitorino et al., 2018; Vargas-Robles et al., 2023; Gosmann et al., 2017; Oliveira de Almeida et al., 2021; Raza et al., 2007). These findings highlight ethnic variability in microbiota composition and its influence on HPV persistence and cancer progression (Martínez et al., 2021).

Dysbiosis in the cervicovaginal microbiome, marked by decreased *Lactobacillus* and increased anaerobic bacteria, fosters a pro-inflammatory environment conducive to HPV persistence and cervical dysplasia (Mitra et al., 2016; Brotman et al., 2014; Doerflinger et al., 2014; Happel et al., 2020; Libertucci and Young, 2019; Marchesi and Ravel, 2015; Ogunrinola et al., 2020; Peebles et al., 2019; Rebersek, 2021; Yang et al., 2021; Zhou et al., 2021). Chronic inflammation driven by cytokines and immune cell recruitment further exacerbates epithelial damage, supporting oncogenesis (Mitra et al., 2015; Shannon et al., 2017; Libby et al., 2008). Microbiota dysbiosis also impairs mucosal barrier function, heightens local inflammation, and promotes conditions for viral persistence and genome integration—key steps in cervical carcinogenesis (Vyshenska et al., 2017; Baldridge et al., 2015; Schneider et al., 2022; Turnbaugh et al., 2007; Wang et al., 2017).

This review explores the association between microbiota, HPV, and cervical cancer by comparing microbial diversity in healthy and HPV-infected women. It highlights the increased prevalence of specific microorganisms in HPV-infected women, such as *Sneathia* spp., *Prevotella*, *Megasphaera*, *Shuttleworthia*, *Streptococcus*, *Porphyromonas*, and *Fusobacterium* spp., and discusses the functional implications of these microbiota shifts. Finally, this review identifies gaps in current research and suggests future directions.

## Methods

We conducted a systematic review on the relationship between cervical microbiota, HPV, and cervical cancer, following PRISMA guidelines. The research question was framed using the PICOS framework. The population included women with HPV infection or cervical cancer, with interventions focusing on cervicovaginal microbiota composition. Outcomes assessed were HPV persistence, microbiota alterations, and cervical cancer progression, with comparators being women with normal microbiota and no HPV.

Advanced searches were conducted in PubMed, EBSCO, and Scopus using predefined search strings combining Medical Subject Headings (MeSH) terms and keywords with Boolean operators (AND, OR). For instance, the PubMed search string was: (“Human papillomavirus” OR “HPV”) AND (“cervical cancer” OR “cervical neoplasia”) AND (“microbiota” OR “microbiome” OR “vaginal microbiome”). Search strings were tailored for each database’s syntax.

References were independently screened to remove duplicates. Titles and abstracts were assessed using predefined inclusion and exclusion criteria. Inclusion criteria required studies on microbiota in women with HPV or cervical cancer, published in English between 2010 and 2024. Exclusion criteria included ongoing studies, pre-prints, qualitative cross-sectional studies, duplicates, and null entries. Relevant details, including author, year, location, study design, patient number, age, and disease description, were extracted. Key findings on cervical microbiome, HPV infection, and cancer progression were analyzed.

**Figure 1:**
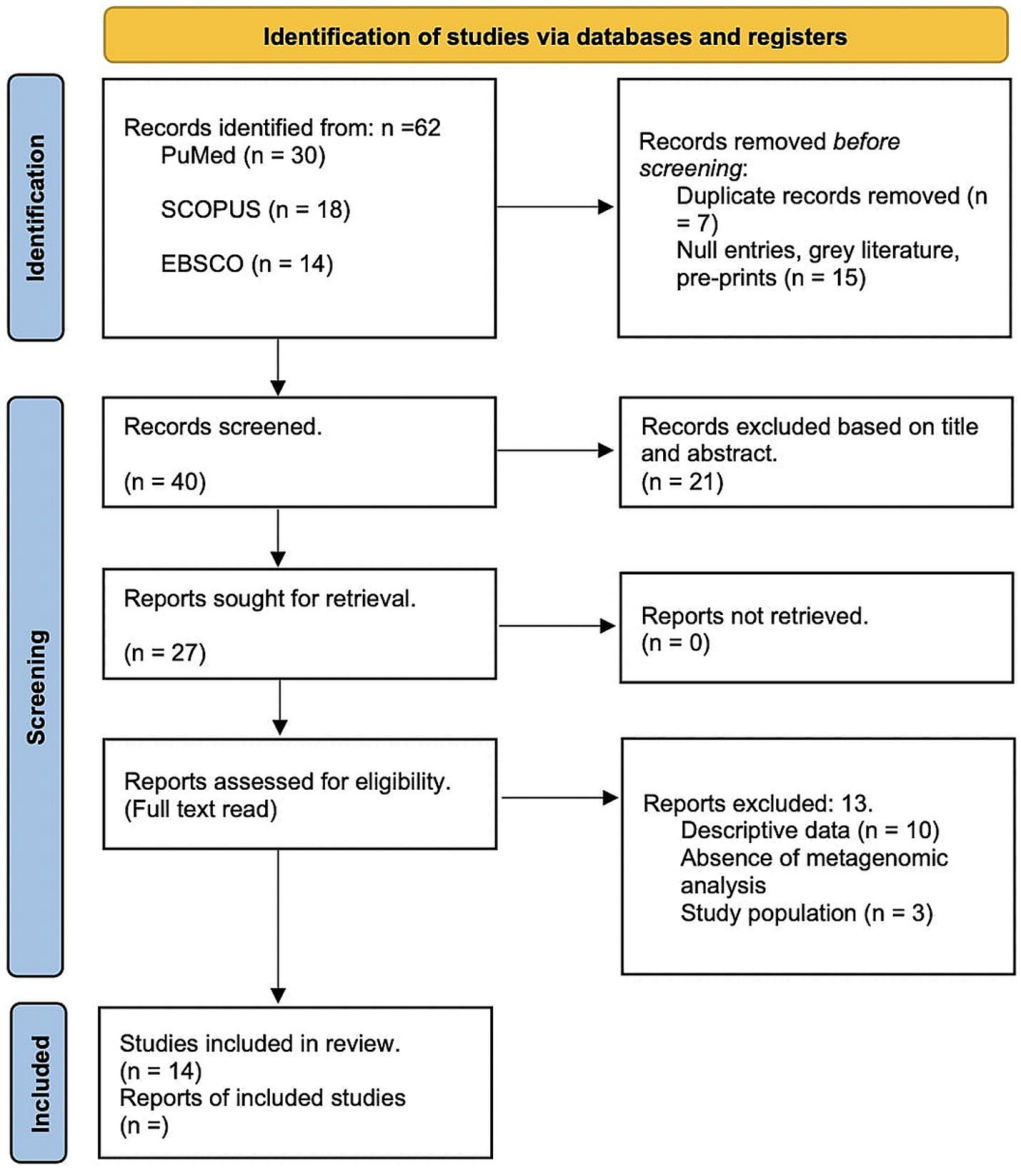
PRISMA Flow Diagram of Study Selection Process. This figure illustrates the PRISMA (Preferred Reporting Items for Systematic Reviews and Meta-Analyses) flow chart detailing the systematic review process for assessing the relationship between cervical microbiota, HPV and cervical cancer progression. The diagram is divided into several sections representing different stages of the review process.

To mitigate bias, the methodological quality of studies was assessed using the Newcastle-Ottawa Scale (NOS), with specific focus on selection, comparability, and outcome assessment domains. Discrepancies between reviewers were resolved through discussion with an external collaborator. A total of 62 papers were identified, with 22 removed as duplicates or irrelevant. Full-text reviews were conducted on 27 studies, of which 14 met the inclusion criteria. The results are outlined in the PRISMA diagram (Figure 1).

### Human papillomavirus

**Figure 2:**
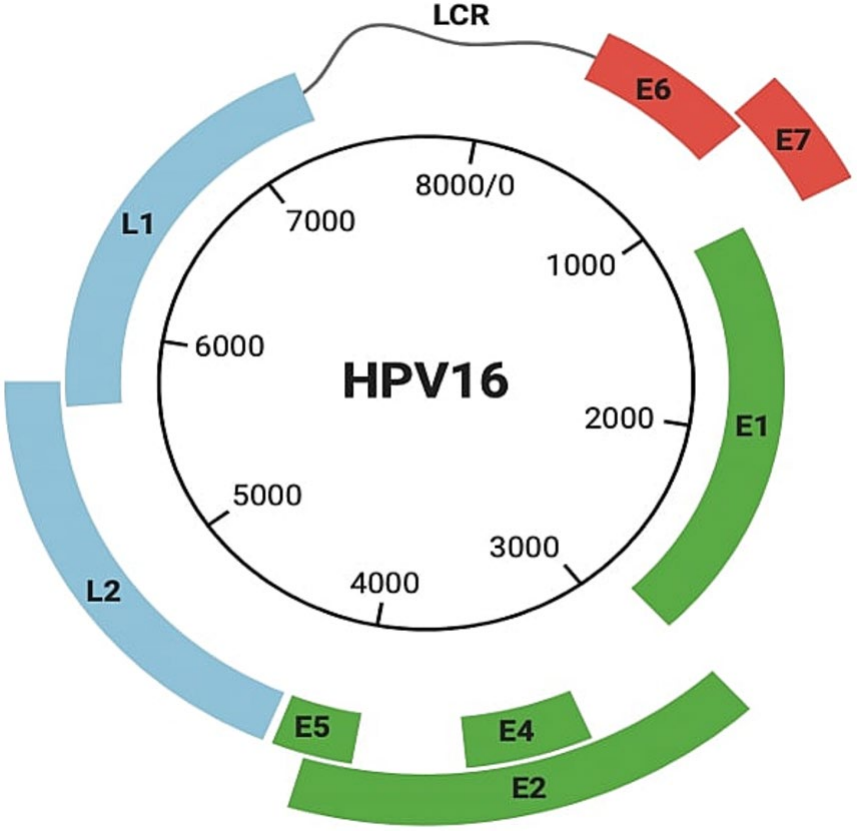
Structure of HPV 16. It includes the noncoding regulatory long control region (LCR), a region containing genes (E1–E7) responsible for transformation and replication, including E6 and E7, and a region encoding capsid proteins L1 and L2.

HPV is a small, non-enveloped, epitheliotropic icosahedral DNA virus (60 nm in diameter) in the subfamily Papillomaviridae and Firstpapillomavirinae. Virions have a single circular double-stranded histone-bound DNA molecule (~8 kb) with eight protein-coding genes (Sharifian et al., 2023). The viral genome has three regions (Figure 2), each contributing to HPV’s ability to infect, replicate, and contribute to carcinogenesis:

1. A noncoding regulatory long control region (LCR) with a promoter, enhancer, and silencer enabling the precise regulation of viral gene expression and replication.
2. A region for transformation and replication, encoding E1 to E7 proteins. The E6 and E7 oncoproteins are particularly significant because they disrupt critical cell cycle regulators, p53 and Rb, promoting uncontrolled cell proliferation. E2, in contrast, plays a regulatory role by downregulating E6 and E7 expression, balancing the viral lifecycle (Brianti et al., 2017; Van Doorslaer et al., 2018).
3. A region encoding capsid proteins L1 and L2, essential for virion assembly (Brianti et al., 2017; Van Doorslaer et al., 2018).

HPV diversity includes 223 different types, with new types continually identified (Bzhalava et al., 2014; de Villiers et al., 2004). Of these, 14 types are high oncogenic risk (HR), including HPV 16 and 18 which are the most frequently associated with cervical carcinogenesis (de Sanjose et al., 2010; Muñoz et al., 2003). Around 71% of cervical carcinoma cases globally involve HPV16 or HPV18, though the prevalence of other genotypes varies by region. For instance, while HPV16 dominates in Europe, HPV52 and HPV58 are more prevalent in parts of Asia, highlighting the geographical variability in HPV genotype distribution and its implications for tailored vaccination strategies (Ye et al., 2024).

### Cervical cancer

Cervical carcinoma is the fourth most prevalent cancer and leading cause of cancer-related mortality among women globally (Fang et al., 2022; Weill Cornell Medicine, n.d.). In 2020, there were 604,127 new cases and 341,831 deaths worldwide (Sharifian et al., 2023; Chang et al., 2023; Siegel et al., 2020). Mortality rates can reach up to 88% in severe cases, particularly in developing countries (Banerjee et al., 2015; Arbyn et al., 2011).

**Figure 3:**
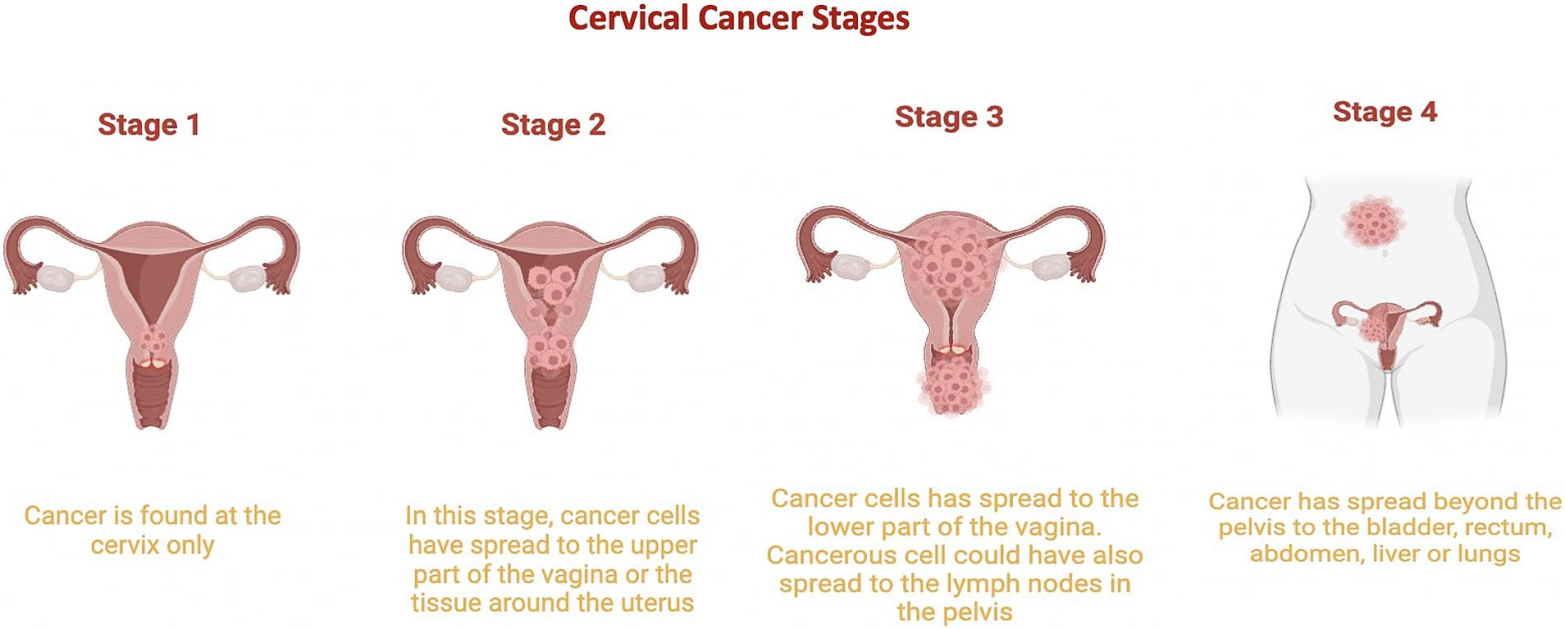
Cervical Cancer Stages. This graphic illustrates the progression of cervical cancer from stage 1, where it is limited to the cervix, to stage 2, where it begins to spread to other regions of the uterus. Stage 3 is characterized by the cancer reaching the lower part of the vagina, and stage 4 shows cancer spreading to other parts of the body. Stage 0 is not included in this depiction.

Cervical cancer involves uncontrolled cell proliferation in the cervix, which connects the uterus to the vagina (National Cancer Institute, 2023). It is classified into five stages (Figure 3):

- Stage 0: Cervical dysplasia, with irregular cells on the cervix surface.
- Stage 1: Cancer confined to the cervix, with tumors 3 mm to 4 cm in diameter.
- Stage 2: Cancer extends beyond the cervix and uterus to the upper two-thirds of the vagina.
- Stage 3: Tumor invades the lower third of the vagina, pelvic walls, and lymph nodes.
- Stage 4: Advanced cancer, with metastasis to distant organs such as the bladder, rectum, liver, lungs, or distant lymph nodes (National Cancer Institute, 2022; American Cancer Society, 2020).

The progression of cervical cancer is closely tied to persistent HPV infection, particularly with high-risk types such as HPV 16 and 18. Persistent HPV infections can lead to cervical dysplasia (Stage 0), a precursor to invasive cervical cancer. As the infection persists, viral oncogenes E6 and E7 disrupt tumor suppressors p53 and Rb, promoting uncontrolled cell proliferation and enabling the transition from precancerous lesions to invasive stages (Figure 3; Doorbar et al., 2012; Crosbie et al., 2013).

### Cervical microbiota

Microbiota refers to the range of commensal, symbiotic, or pathogenic microorganisms found in multicellular organisms. Each part of the human body has a specific microbiota playing a key role in health, such as the intestinal or vaginal microbiota (Sharifian et al., 2023). The disruption of microbiota homeostasis increases vulnerability to viral infections (Avilés-Jiménez et al., 2017; Schwabe and Jobin, 2013). Changes in the abundance of certain microorganisms, their functional abilities and the changes caused in the metabolic pathways are factors that can contribute to cancer progression (Shin et al., 2015; Heintz-Buschart and Wilmes, 2018; Deng et al., 2021). Yet, the exact mechanisms of how microbiota disruptions lead to diseases are still unknown (Condic et al., 2023).

The role of the microbiome in cancer development has gained recognition, now considered one of the emerging hallmarks of cancer (Hanahan, 2022; Ekström et al., 2013). Microbial communities influence processes like inflammation, immune evasion, and metabolic reprogramming.

The vaginal microbiota thrives in an anaerobic habitat, receiving nutrients like glucose and oxygen (Linhares et al., 2011). It is also dynamic, influenced by age, menstrual cycle, sexual activity, stress, and pregnancy (Chen et al., 2021; Culhane et al., 2002; Noyes et al., 2018; Aagaard et al., 2012). Vaginal dysbiosis, the most common disorder among reproductive-age women, involves irregular microbial growth, increased diversity, and imbalance, leading to higher infection susceptibility (Sharifian et al., 2023; Chen et al., 2021; Javed et al., 2019; Eschenbach, 1993) with symptoms such as elevated vaginal pH, irritation and discharge (Torcia, 2019; Brotman et al., 2014). Dysbiosis contributes to cervical cancer development through epithelial barrier disruption, metabolic dysregulation, abnormal cell proliferation, genome instability, chronic inflammation, and angiogenesis (Sharifian et al., 2023; Castanheira et al., 2021; Mitra et al., 2016).

Microbial metabolism plays a crucial role in modulating the cervicovaginal microenvironment and promoting carcinogenesis. Dysbiotic microbiota alter metabolite production, such as short-chain fatty acids (SCFAs), which typically support vaginal health but can promote inflammation under dysbiosis (Tjalsma et al., 2012; Bokulich et al., 2022). Elevated SCFA levels activate pro-inflammatory pathways like NF-κB, increasing cytokines such as IL-6 and TNF-*α*, creating a pro-carcinogenic environment (Mitra et al., 2016).

Inflammatory cytokines like IL-1β, IL-6, and IL-8, elevated during dysbiosis, recruit immune cells that release reactive oxygen species (ROS), causing oxidative stress and DNA damage. This facilitates HPV genome integration, overexpressing E6 and E7 oncoproteins, which drive cellular proliferation and inhibit apoptosis (Schmitt et al., 1994; Sharifian et al., 2023).

Pathways such as glycan biosynthesis and amino acid metabolism, enriched in dysbiotic microbiomes, further promote cancer progression. Glycan biosynthesis weakens the epithelial barrier, increasing pathogen invasion (Fang et al., 2022), while amino acid metabolism supports pathogenic bacteria growth, exacerbating inflammation and HPV persistence (Usyk et al., 2020).

Vaginal microbes are classified into 5 community status types (CST) based on the predominance of certain species. The CST classification system was originally proposed by Ravel et al. (2011), identifying CST-I, CST-II, CST-III, and CST-V are dominated by *Lactobacillus* species: *L. crispatus, L. gasseri, L. iners, and L. jensenii*, respectively. CST-IV is divided into CST IV-A, with modest *Lactobacillus* presence, and CST IV-B, dominated by anaerobes like *Atopobium, Prevotella, Parvimonas, Gardnerella, and Megasphera* (Chen et al., 2021; Sharifian et al., 2023; Kyrgiou et al., 2017; Romero et al., 2014; Table 1). Recently, the CST classification has been refined using VALENCIA software, which identifies CSTs based on amplicon sequencing data and provides a more standardized approach to classifying vaginal microbial communities. Unlike the original framework, VALENCIA offers greater granularity, particularly within CST-IV, by identifying subtypes dominated by specific anaerobes, thus enabling a more detailed understanding of dysbiotic states (Gajer et al., 2012; France et al., 2020).

**Table 1:** Summary of studies on cervical microbiota composition on HPV and cervical cancer women.

Study	Key Findings	Methodology	Limitations	Country
Chen et al. (2021)	Healthy women show lower microbiota diversity and higher presence of *Lactobacillus* spp.; Women with bacterial vaginosis show higher diversity and presence of anaerobic bacteria	C comprehensive literature review with predefined criteria and data extraction	High heterogeneity across studies; did not deeply analyze functional implications of microbiota changes	Various (literature review)
Muzny et al. (2018)	Healthy cervical microbiota characterized by *Lactobacillus* spp.; Increase in *Gardnerella vaginalis, Prevotella bivia, and Atopobium vaginae* linked to dysbiosis	Self-collected samples, specific population (African American women who have sex with women)	Variability in sample quality and timing; limited generalizability to other populations	United States
Srinivasan et al. (2012)	Women with bacterial vaginosis have higher bacterial diversity, predominantly anaerobes like *Gardnerella vaginalis, Atopobium vaginae, and Mobiluncus*	High-resolution phylogenetic analyses	None mentioned	United States
Marconi et al. (2020)	*Lactobacillus* (specifically *L. crispatus*) dominant in HPV-negative women; crucial for maintaining vaginal health by producing lactic acid	Sequenced cervical samples from over 500 women from five different regions in Brazil	Focused on taxonomic composition, not functional implications	Brazil
Motevaseli et al. (2016)	*L. crispatus* culture supernatants decreased HPV E6 oncogene expression, creating an anti-proliferative state	Culturing HeLa cells treated with culture supernatants of *L. crispatus*	In vitro experiments may not replicate in vivo conditions; focused on a few autophagy genes	Iran
Lee et al. (2013)	HPV-positive women had more diverse vaginal microbiota; higher proportions of anaerobic bacteria like *Gardnerella, Atopobium, and Prevotella*	Study on a Korean twin cohort	Findings specific to Korean twin cohort; small sample sizes for some subgroup analyses	South Korea
So et al. (2020)	Cervical cancer associated with increased anaerobic bacteria like *Atopobium and Prevotella, especially Gardnerella*	Investigated changes in vaginal microbiota during cervical carcinogenesis in women with HPV	Small sample size (only 50 women, 10 healthy); limited generalizability	South Korea
Chen et al. (2020)	*Gardnerella* present in HPV-negative women at lower levels; significantly higher in HPV-positive women	Analysis of Gardnerella and other anaerobic bacteria in relation to HPV infection and cervical intraepithelial neoplasia progression	None mentioned	China
Tango et al. (2020)	*Gardnerella* can be present in HPV-negative women but significantly increases in HPV-positive individuals and those with cervical cancer	Study on taxonomic and functional differences in cervical microbiota associated with cervical cancer	None mentioned	South Korea
Sharifian et al. (2023)	Increased diversity in vaginal microbiota, higher presence of anaerobic bacteria like *Prevotella* linked to HPV infection and cancer progression	Comprehensive review of literature on microbiota diversity and its impact on HPV and cancer progression	None mentioned	Various (literature review)

This table summarizes key information from various studies examining the relationship between cervical microbiota, HPV, and cervical cancer. It includes the main findings, methodologies used, any limitations noted in each study, and the countries where the studies were conducted. This comprehensive overview highlights the significant role of microbial diversity and specific bacterial species in the progression and management of cervical cancer.

### Metagenomics of the vaginal microbiome during HPV infection

Recent years have seen a revolution in studying microbiota and their connection to cancer with the advent of metagenomics (Banerjee et al., 2015). Metagenomics examines the functions, structures, and interactions of microorganisms by analyzing entire nucleotide sequences from bulk samples (Banerjee et al., 2015). Previously, microbiota studies relied on traditional bacterial culture methods, which were limited because most microorganisms cannot be cultured in laboratories (Fang et al., 2022; Arokiyaraj et al., 2018; Wei et al., 2021). Using whole metagenomic sequencing, researchers can sequence the entire DNA within a sample, increasing the depth and specificity of identified species and providing insights into gene function and metabolic pathways (Fang et al., 2022; Shah et al., 2018; Biegert et al., 2021). This approach has enabled associations between the predominance of certain microorganisms in the vaginal microbiome and the development of cervical cancer (Luan et al., 2020).

It is well established that healthy women tend to show lower microbiota diversity and a higher presence of *Lactobacillus* spp. compared to women with bacterial vaginosis. For instance, Chen et al. (2021) review article highlights the differences in the vaginal microbiome between healthy women and those with bacterial vaginosis showing that in healthy women, the vaginal microbiome is predominantly composed of *Lactobacillus* spp. For this, they compiled information by conducting a thorough literature search, selecting relevant studies based on predefined criteria, extracting, and analyzing key data, and integrating findings to provide a comprehensive review of the female vaginal microbiome. Studies claim that healthy cervical microbiota is characterized by the presence of *Lactobacillus* spp. whereas an increase in *Gardnerella vaginalis*, *Prevotella bivia*, and *Atopobium vaginae*, is significantly associated with the development of dysbiosis (Muzny et al., 2018). One caveat of the study is that results rely on self-collected samples could introduce variability in sample quality and timing. Additionally, the study focused on a specific population, African American women who have sex with women, which may limit the generalizability of the findings to other groups. Fredricks et al. (2007) also argue that the presence of certain bacteria, such as *Gardnerella vaginalis*, *Atopobium vaginae*, and *BV-associated bacterium-1*, was strongly associated with bacterial vaginosis. It is, however, important to notice that in the study they used targeted PCR assays for the detection of vaginal bacteria in vaginal samples collected from women diagnosed with vaginal dysbiosis which could lead to some bacteria missing from the essay. Finally, the study by Srinivasan et al. (2012) found significant differences in the bacterial communities of healthy women compared to those with bacterial vaginosis. Using high-resolution phylogenetic analyses, the study identified that women with vaginosis had a higher diversity of bacterial species, predominantly anaerobes such as *Gardnerella vaginalis*, *Atopobium vaginae*, and *Mobiluncus*.

Healthy cervical microbiota is overrepresented with *Lactobacillus* spp. since they can withstand infections by producing bacteriocin, biosurfactants, and lactic acid (Banerjee et al., 2015; Fang et al., 2022; Witkin and Linhares, 2017). For example, a study in Brazil sequenced cervical samples from over 500 women from five different regions and found that *Lactobacillus*, specifically *L. crispatus*, is the dominant species in HPV-negative women. They argue *Lactobacillus* spp. are crucial for maintaining vaginal health by producing lactic acid and maintaining a low pH environment (Marconi et al., 2020). Yet, it is important to acknowledge that, the study focused on taxonomic composition without delving into the functional implications of the microbiota, which could provide deeper insights into the health impacts of microbiota changes. The study by Chen et al. (2021) demonstrated, using 16S rRNA gene sequencing, that in healthy women, the vaginal microbiome is predominantly composed of *Lactobacillus* spp., including species such as *Lactobacillus crispatus* and *Lactobacillus iners*. These bacteria produce antimicrobial compounds like lactic acid and bacteriocins, which maintain a low vaginal pH and inhibit pathogen colonization. In contrast, the cervical microbiota of women with vaginal dysbiosis, caused by HPV, is characterized by a marked reduction in *Lactobacillus* spp. and an increase in anaerobic bacteria, including *Gardnerella vaginalis*, which forms biofilms and shelters other bacterial vaginosis-associated microbes. One study’s limitation is that it acknowledges the high heterogeneity across different studies, making it challenging to generalize findings to other settings. This study also does not delve deeply into the functional implications of the microbiome composition changes which could further explain the importance of *Lactobacillus* spp. in healthy women. Furthermore, a study culturing HeLa cells treated with culture supernatants of *Lactobacillus crispatus* showed a significantly decreased the expression of the HPV E6 oncogene, creating an anti-proliferative state (Motevaseli et al., 2016). However, the study’s findings are based on *in vitro* experiments, which may not fully replicate *in vivo* conditions and only focused on a few autophagy genes, leaving out potential impacts on other relevant pathways or genes. Results are also specific to HeLa cells and may not be generalizable to other cell types.

While Lactobacillus dominance is generally protective for vaginal health, specific species play varied roles. *Lactobacillus crispatus* is the most protective, producing high levels of lactic acid to maintain low vaginal pH, inhibit pathogens, and strengthen the epithelial barrier (Petrova et al., 2017; Borges et al., 2022). Its production of bacteriocins and hydrogen peroxide further enhances its protective effects.

Conversely, *Lactobacillus iners* is associated with transitional microbiota states. It adapts to dysbiotic environments and may contribute to inflammation through its enzyme and metabolite production, potentially promoting HPV persistence and cervical dysplasia (Vaneechoutte, 2017; van der Veer et al., 2017). *Lactobacillus jensenii* and *Lactobacillus gasseri* have intermediate roles, with lower acid production and less pronounced pathogen inhibition compared to *L. crispatus*.

Based on this information it is not surprising that CST-I and CST-II are common in HPV-negative women, while CST-IV dominates during HPV infection and cervical cancer development (Table 2; Chen et al., 2021; Brotman et al., 2014; Shannon et al., 2017; Xu et al., 2020). CST-IV is associated with persistent HPV due to *G. vaginalis* secreting vaginolysin, causing cellular lysis and dysbiosis (Sharifian et al., 2023; Nowak et al., 2018). CST-III characterized by a dominant presence of *L. iners* is prevalent among HPV-positive women because this bacteria can survive in varying pH ranges and inhibits pathogen colonization (Sharifian et al., 2023; Romero et al., 2014; Macklaim et al., 2013; Macklaim et al., 2011). *L. iners* also produces inerolysin, a cytotoxin that creates pores, facilitating HPV entry into the vaginal epithelium (Sharifian et al., 2023; Pleckaityte, 2020; Curty et al., 2019).

**Table 2:** Dominant species in vaginal community state types (CSTs).

Vaginal community state types	Dominant species
CST I	*Lactobacillus crispatus*
CST II	*Lactobacillus gasseri*
CST III	*Lactobacillus iners*
CST IVA	Dominated by *Candidatus Lachnocurva vaginae* but also presence of *Gardnerella vaginalis, Atopobiumvaginae and Prevotella species*
CST IVB	Dominated by *Gardnerella vaginalis,* but also presence of *Candidatus Lachnocurva vaginae, Atopobium vaginale and Prevotella species*
CST V	*Lactobacillus jensenii*

This table lists the dominant species corresponding to each of the five categories of vaginal community state types. For CST IV, the table includes the two subdivisions (IV-A and IV-B) and specifies the most common species found in each.

As HPV infection progresses, the abundance of *Lactobacillus* spp. decreases and is accompanied by a sharp increase in diversity of anaerobic bacteria such as *Sneathia* spp. and *Fusobacterium* spp. (Aitmanaitė et al., 2023; Audirac-Chalifour et al., 2016; Łaniewski et al., 2018; Wu et al., 2021; Tango et al., 2020). For example, the study by Lee et al. (2013) showed that HPV-positive women had a more diverse vaginal microbiota compared to HPV-negative women in a Korean twin cohort. The dominant bacterial genera in HPV-negative women were *Lactobacillus species*, whereas HPV-positive women exhibited higher proportions of anaerobic bacteria such as *Gardnerella, Atopobium, and Prevotella*. While these findings provide valuable insights into the microbiota-HPV connection, the study was conducted exclusively on a Korean cohort, which limits the generalizability of the results to other populations, as each population has unique microbiota characteristics influenced by genetic, environmental, and cultural factors. Nonetheless, it serves as an important reference for understanding the relationship between HPV status and microbiota diversity. Some subgroup analyses, such as those involving postmenopausal women, had relatively small sample sizes, affecting the robustness of the conclusions.

The presence of *Gardnerella* could be used as a potential marker for HPV infection and cancer progression. Some studies argue that *Gardnerella* is mainly found in women infected with HPV and cervical cancer (Lee et al., 2013). In fact, a study by So et al. (2020) that investigated the changes in vaginal microbiota during cervical carcinogenesis in women with HPV infection indicated that women with cervical cancer showed an increase in anaerobic bacteria such as *Atopobium, and Prevotella* and specially *Gardnerella.* However, they sampled only 50 women with only 10 of them being healthy. Thus, the relatively small sample size may affect the generalizability of the findings. According to a study by Chen et al. (2020), *Gardnerella* was present in HPV-negative women, although at lower levels compared to HPV-positive women. This highlights that the presence and abundance of *Gardnerella* and other anaerobic bacteria increase significantly in women with HPV infection and cervical intraepithelial neoplasia progression. A study by Tango et al. (2020) also indicates that *Gardnerella* can be present in HPV-negative women, but its abundance significantly increases in HPV-positive individuals and those with cervical cancer. While *Gardnerella* is frequently associated with HPV infection and cervical cancer (Brotman et al., 2014; Mitra et al., 2016), its reliability as a biomarker is limited due to low specificity and its presence in other dysbiotic conditions like bacterial vaginosis. Rather than serving as a standalone marker, *Gardnerella* is better understood as part of a dysbiotic microbial community contributing to disease progression. Further studies are needed to quantify its sensitivity, specificity, and predictive values for clinical use.

*Prevotella* is another genus frequently linked to cervical cancer progression, as it provides nutrients to other dysbiosis-related bacteria, making the host more vulnerable to HPV infections (Sharifian et al., 2023; Pybus and Onderdonk, 1997; Chao et al., 2020). Sharifian et al. (2023) reported that increased vaginal microbiota diversity, particularly a higher presence of anaerobic bacteria such as *Prevotella*, is associated with HPV infection and progression to cervical cancer. However, *Prevotella* is also commonly found in other dysbiotic conditions, such as bacterial vaginosis and pelvic inflammatory disease, raising questions about its specific role in cervical carcinogenesis (Mitra et al., 2016; Brotman et al., 2014). Pybus and Onderdonk (1997) demonstrated a symbiotic relationship between *Gardnerella vaginalis* and *Prevotella bivia*, where ammonia produced by *P. bivia* supports the growth of *G. vaginalis*. This interaction may contribute to bacterial vaginosis pathogenesis by promoting an environment conducive to overgrowth of BV-associated bacteria, which could indirectly influence HPV persistence and disease progression.

In addition to *Gardnerella* and *Prevotella*, other anaerobic and facultative anaerobic bacteria, such as *Sneathia*, *Megasphaera*, *Streptococcus*, and *Fusobacterium* spp., play significant roles in HPV persistence and cervical cancer development. *Sneathia* spp. have been consistently associated with cervical dysplasia and cancer, likely due to their ability to induce pro-inflammatory cytokines and damage epithelial barriers, creating a microenvironment conducive to HPV persistence and integration into the host genome (Mitchell et al., 2015; Brotman et al., 2014). Similarly, *Megasphaera* spp. are known to produce metabolites that disrupt vaginal pH and promote immune evasion, facilitating viral persistence and progression to neoplasia (Mitra et al., 2016). *Fusobacterium* spp., commonly implicated in other cancers such as colorectal cancer, have also been identified in cervical dysplasia and cancer. They may contribute to carcinogenesis through chronic inflammation, production of genotoxic metabolites, and interactions with host immune cells that suppress anti-tumor responses (Kostic et al., 2013; Shannon et al., 2017). Additionally, while *Streptococcus* spp. are not traditionally associated with vaginal dysbiosis, certain pathogenic strains have been linked to HPV persistence through their potential to enhance inflammation and alter mucosal immunity, further supporting a permissive environment for viral oncogenesis (Gillet et al., 2011).

Co-infections with other sexually transmitted infections (STIs), such as *Chlamydia trachomatis*, *Neisseria gonorrhoeae*, and *Trichomonas vaginalis*, have been shown to exacerbate HPV infection and persistence, potentially contributing to cervical cancer progression. These pathogens induce chronic inflammation and disrupt the epithelial barrier, creating an environment conducive to HPV replication and persistence (Gillet et al., 2011; Moscicki et al., 2012). For example, *Chlamydia trachomatis* has been associated with increased expression of pro-inflammatory cytokines, which may impair HPV clearance and promote oncogenesis (Moscicki et al., 2012). While not the focus of this review, understanding the interplay between HPV and co-infecting STIs highlights the importance of addressing these co-factors in cervical cancer prevention strategies.

Women who receive the HPV vaccine are largely protected from cancer caused by high-risk HPV strains. Limited evidence suggests that vaccination does not directly alter the cervicovaginal microbiota but may indirectly affect it by reducing HPV-induced dysbiosis. For example, studies have shown that HPV infection is associated with shifts in microbiota composition, including reduced *Lactobacillus* dominance and increased anaerobic bacteria such as *Gardnerella* and *Prevotella* (Mitra et al., 2016; Brotman et al., 2014). By preventing HPV infection, vaccination may help maintain a more stable and protective microbiota.

### Functional role of microbiota vaginal during cervical cancer

**Figure 4:**
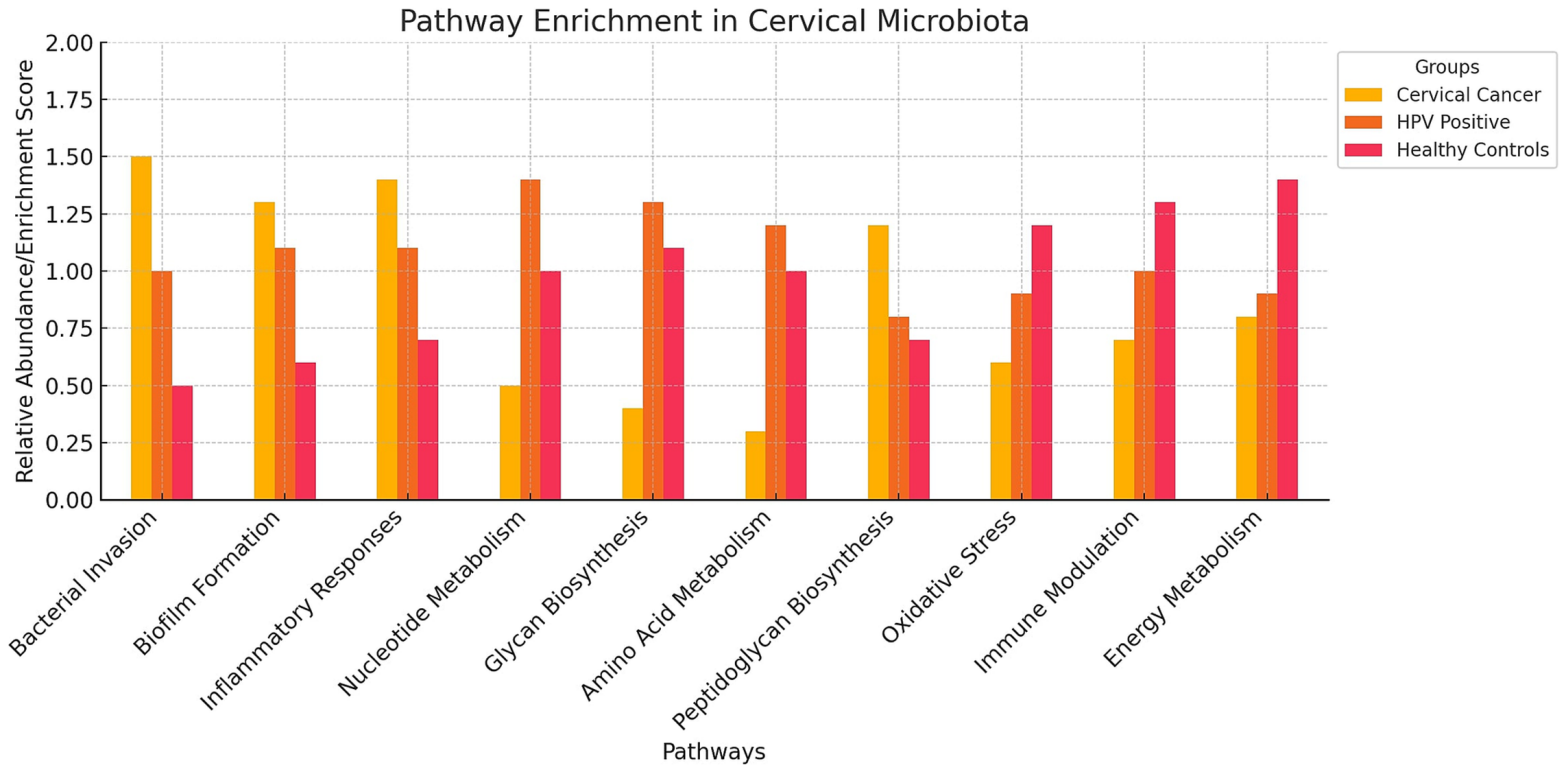
Pathway Enrichment Bar Chart on Cervical Microbiota. The bar chart visualizes the relative abundance of various pathways in the cervical microbiota across three different groups: cervical cancer patients, HPV-positive women, and healthy controls. The chart highlights that cervical cancer patients (red bars) show higher enrichment in pathways related to bacterial invasion, biofilm formation, and inflammatory responses. HPV-positive women (blue bars) exhibit increased activity in nucleotide metabolism, glycan biosynthesis, and amino acid metabolism. Healthy controls (green bars) have lower enrichment scores in pathways associated with cancer and viral infection.

Metagenomic studies also allow us to explore the functional profile of genes annotated during the sequencing analysis. For example, Kwon et al. (2019) used the KEGG database for pathway annotation and the HUMAnN2 pipeline to map microbial genes to metabolic pathways, identifying enriched pathways in cervical cancer patients compared to healthy women. These included bacterial invasion, biofilm formation, and inflammatory responses, highlighting how altered microbiota promotes bacterial persistence, immune evasion, and chronic inflammation, all contributing to carcinogenesis (Table 3; Figure 4). However, it is important to note that these findings do not establish causality, as microbiota alterations may also result from carcinogenesis rather than act as a direct causal factor.

**Table 3:** Summary of enriched and depleted pathways/functions on cervical cancer and microbiome studies.

Study	Enriched pathways	Depleted pathways/functions
Kwon et al. (2019)	Bacterial invasion, biofilm formation, inflammatory responses, peptidoglycan synthesis	Dioxin degradation, 4-oxalocrotonate tautomerase activity, COG category related to ‘Defense mechanisms’
Tango et al. (2020)	Bacterial invasion, biofilm formation, inflammatory responses	Not specified
Fang et al. (2022)	Nucleotide metabolism, glycan biosynthesis, amino acid metabolism	Not specified
Usyk et al. (2020)	Nucleotide metabolism, glycan biosynthesis, amino acid metabolism	Not specified
Karlsson et al. (2012)	Peptidoglycan synthesis	Not specified (focused on gut microbiome in atherosclerosis, analogy to cervical cancer discussed)

This table shows the pathways and functions that were found to be enriched or not enriched in various studies investigating the functional profiles of cervical microbiota in relation to HPV and cervical cancer. It includes the main findings from each study, notes any limitations, and provides context on the specific pathways and functions investigated.

Similarly, Tango et al. (2020) used KEGG-based functional analyses and shotgun sequencing to confirm the enrichment of these pathways in cervical cancer patients (Table 3). Their findings reinforce the role of chronic inflammation and microbial persistence in disease progression, though the lack of temporal data restricts understanding of dynamic microbiota changes.

Fang et al. (2022) and Usyk et al. (2020) applied KEGG annotations and HUMAnN2, identifying enriched pathways for nucleotide metabolism, glycan biosynthesis, and amino acid metabolism in HPV-positive women (Table 3; Figure 4). These functional enrichments support viral replication, epithelial barrier disruption, and an environment conducive to prolonged HPV infection.

Pathway enrichment also revealed upregulated peptidoglycan biosynthesis in cervical cancer patients (Kwon et al., 2019), which may sustain bacterial populations driving inflammation. Karlsson et al. (2012) similarly found enriched peptidoglycan biosynthesis in gut microbiota associated with atherosclerosis, suggesting shared inflammatory mechanisms. Additionally, Kwon et al. (2019) observed depletions in pathways for dioxin degradation and defense mechanisms in cervical cancer patients (Table 4; Figure 4), indicating reduced toxin processing and weakened microbial defense, which may exacerbate HPV persistence. Small sample sizes and population specificity highlight the need for validation in diverse cohorts.

**Table 4:** Summary of studies on cervical microbiota functionality and its impact on cervical cancer progression.

Study	Key findings	Methodology	Limitations	Country
Kwon et al. (2019)	Increased pathways related to bacterial invasion, biofilm formation, and inflammatory responses in cervical cancer microbiota. Suggests functional changes may contribute to carcinogenesis.	Shotgun sequencing of cervical samples	Conducted on a specific population, limiting generalizability	South Korea
Tango et al. (2020)	Significant functional differences in cervical microbiome associated with cancer. Enriched bacterial invasion, biofilm formation, and inflammation pathways. Indicates microbiome-induced inflammation as a factor.	Functional analysis of cervical microbiomes	Did not account for temporal changes in the microbiome	South Korea
Fang et al. (2022)	HPV-positive women showed increased pathways related to nucleotide metabolism, glycan biosynthesis, and amino acid metabolism, supporting viral replication and persistence.	Metagenomic sequencing of cervical samples	Focused on functional analysis without deep taxonomic analysis	China
Usyk et al. (2020)	Enriched pathways in nucleotide metabolism, glycan biosynthesis, and amino acid metabolism in HPV-positive women.	Longitudinal study with metagenomic analysis	Lack of temporal resolution in microbiome changes	Costa Rica
Karlsson et al. (2012)	Enrichment in peptidoglycan synthesis pathways in patients with symptomatic atherosclerosis. Suggests potential role in inflammatory responses and disease progression.	Metagenomic analysis of gut microbiomes	Conducted on fecal samples, which may not directly correlate with vaginal microbiome	Sweden

Table summarizes key findings, methodologies, limitations, and countries of studies exploring the functional profiles of cervical microbiota in relation to HPV and cervical cancer. It highlights the significant pathways and microbial functions that are altered in cervical cancer patients compared to healthy individuals. The table also notes the limitations of each study, such as the specific populations studied and the methodologies used, which may impact the generalizability of the findings.

## Future perspectives

Recent studies provide evidence of an association between cervical microbiota composition and cervical cancer development, though causal relationships remain to be validated through prospective cohort or experimental studies (Usyk et al., 2020; Kwon et al., 2019; Fang et al., 2022). However, the mechanisms underlying this relationship remain unclear. This is mainly because studies exploring the role of cervical microbiota in cancer development are scarce due to significant challenges. Longitudinal studies require large cohorts and extended follow-up periods to capture the temporal dynamics of microbiota changes, which demand considerable resources and logistical coordination (Shannon et al., 2017; Usyk et al., 2020). Functional metagenomics and host-microbiome interaction studies necessitate advanced bioinformatics tools and experimental validation, which are both time-intensive and costly (Fang et al., 2022; Kwon et al., 2019). External factors such as diet and antibiotic use introduce variability that is difficult to control in human populations, further complicating study design (Brotman et al., 2014). These complexities highlight why such research is limited, despite its critical importance in understanding the microbiota’s role in cancer progression.

**Figure 5:**
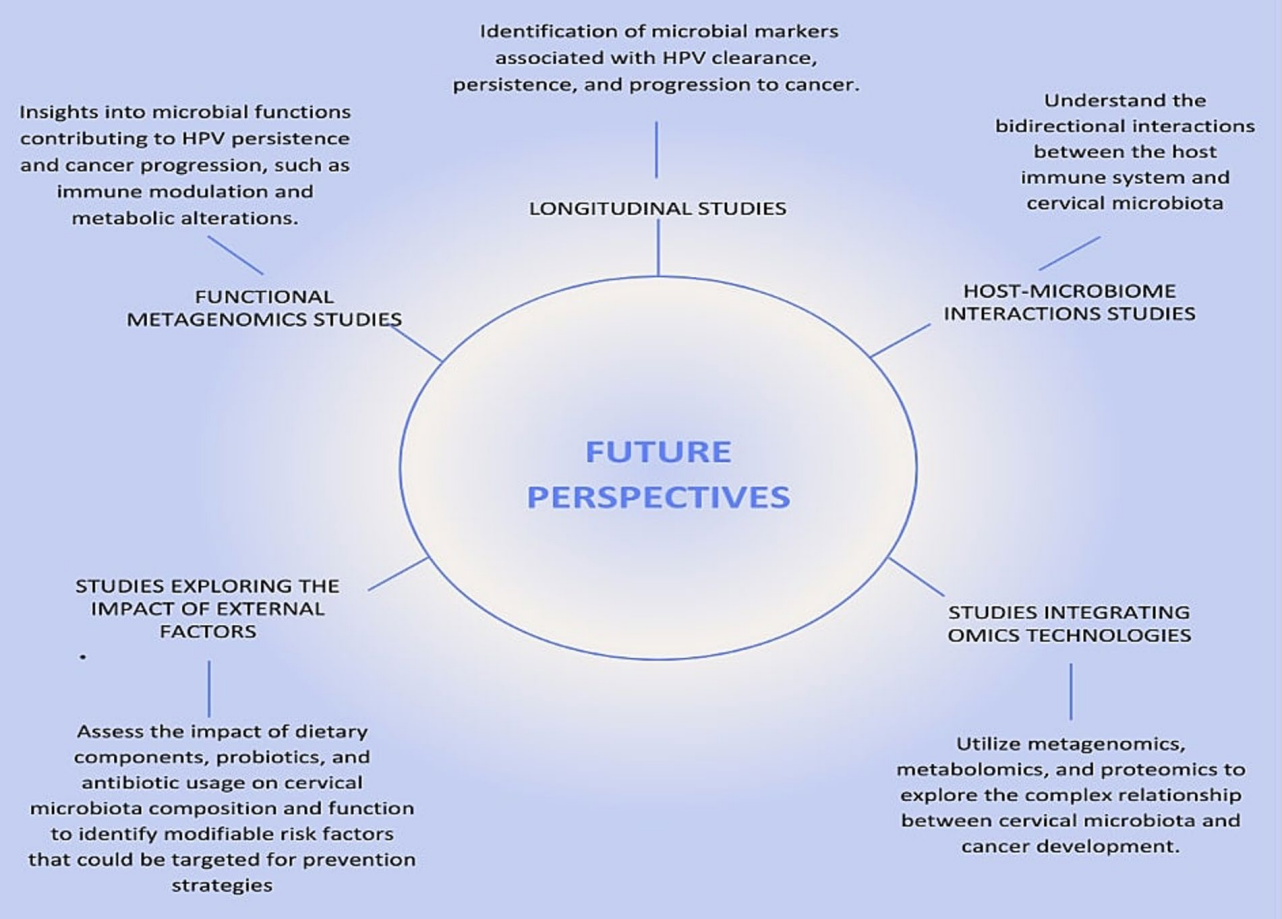
Future Perspectives on the Relationship Between HPV, Cervical Cancer, and Microbiota. This figure illustrates the key future research directions necessary to deepen our understanding of the relationship between human papillomavirus (HPV), cervical cancer, and cervical microbiota. The central node, labeled “Future Research Directions,” connects to five critical areas of study, each represented by a radial node.

Longitudinal studies will enable to investigate the temporal relationship between changes in cervical microbiota and the progression of HPV infections to cervical cancer. These studies offer vital insights into how microbiota composition and functional pathways change during infection and disease progression. Large-scale longitudinal cohort studies with regular cervical microbiota and HPV sampling are essential to identify microbial markers linked to HPV clearance, persistence, and cancer progression (Figure 5). For instance, Usyk et al. (2020) stressed the value of longitudinal data but was limited by only two sampling points, restricting its ability to track microbiome dynamics over time. Recent studies by Shannon et al. (2017) and Huang et al. (2022) provide deeper insights into temporal microbiota-HPV interactions. Shannon et al. found fluctuations in *Lactobacillus* spp. dominance associated with transitions between HPV persistence and clearance, emphasizing frequent sampling to capture microbiome shifts. Similarly, Huang et al. linked temporal increases in anaerobes like *Prevotella* and *Gardnerella* to HPV persistence and progression to high-grade cervical lesions, highlighting the dynamic microbiome changes during disease development.

Functional metagenomics will allow the exploration of the functional capabilities of the cervical microbiota and their role in modulating the host environment and immune response. While most studies rely on 16S rRNA sequencing, which provides valuable taxonomic information (Ranjan et al., 2016), tools such as PiCrust2 extend its utility by inferring metabolic pathways from 16S data. For instance, a study by France et al. (2021) used PiCrust2 to analyze the vaginal microbiome in HPV-positive and HPV-negative women, identifying functional pathways associated with immune modulation and inflammation, such as increased nucleotide metabolism and glycan biosynthesis, in HPV-positive individuals. Similarly, Zhang et al. (2020) employed PiCrust to infer functional gene profiles of vaginal microbiomes, linking dysbiotic microbiota with enhanced pro-inflammatory pathways, highlighting their potential role in HPV persistence and progression to cervical cancer. These applications demonstrate that PiCrust2 is a powerful tool for generating hypotheses and gaining functional insights into microbial communities using 16S data. However, because these inferences are based on predicted gene content rather than direct measurements, they lack the precision and depth provided by shotgun metagenomic sequencing. Shotgun approaches can identify the full spectrum of microbial genes and metabolic pathways, including previously uncharacterized ones, offering new insights into how microbial functions may contribute to immune modulation and metabolic alterations that drive HPV persistence and cancer progression (Figure 5). Integrating both approaches could therefore provide a more comprehensive understanding of the microbiome’s role in cervical carcinogenesis.

Emerging evidence suggests that the cervicovaginal microbiota modulates immune responses through metabolite production and inflammatory cytokines (Aggarwal et al., 2023; Cullin et al., 2021). Bokulich et al. (2022) identified microbial metabolites as key predictors of the cervicovaginal microenvironment, influencing immune responses and contributing to HPV persistence and carcinogenesis. Similarly, Tosado-Rodríguez et al. (2024) showed that dysbiotic microbiota, characterized by increased microbial diversity and reduced *Lactobacillus* spp., is linked to elevated inflammatory cytokines in women with cervical dysplasia. These cytokines exacerbate chronic inflammation, disrupt epithelial integrity, and facilitate HPV immune evasion.

Further research into host-microbiome interactions is needed to explore how microbial communities influence immune responses and vice versa. Techniques such as host transcriptomics and microbiome profiling in women with varying HPV and cervical cancer statuses are recommended (Kwon et al., 2019).

External factors, including diet and antibiotics, also affect cervical microbiota and cancer risk. High-fat, low-fiber diets have been linked to altered vaginal microbiota, promoting inflammation and HPV persistence (Piyathilake et al., 2016; Kim et al., 2020). Overuse of antibiotics disrupts microbial diversity and reduces protective *Lactobacillus* populations, increasing dysbiosis and HPV persistence (Sobel et al., 2019; Macklaim et al., 2015). Studies exploring the impact of diet, probiotics, and antibiotics on cervical microbiota are essential to identify modifiable risk factors (Figure 5). For example, Zamani et al. (2014) demonstrated how diet and antibiotics alter gut microbiota and metabolic profiles in colon cancer patients, identifying potential biomarkers using nuclear magnetic resonance (NMR) spectroscopy. However, combining NMR with mass spectrometry could enhance metabolite detection.

Omics technologies, including metagenomics, metabolomics, and proteomics, offer comprehensive tools to explore the relationship between cervical microbiota and cancer development. These approaches detail microbial communities and their functional roles, revealing how microbiota-driven metabolic alterations contribute to carcinogenesis (Zamani et al., 2014). Metabolic pathways such as nucleotide metabolism, glycan biosynthesis, and amino acid metabolism have been linked to HPV infections and cervical cancer progression (Fang et al., 2022). Proteomics analyzes protein expressions and modifications, uncovering host immune responses and molecular mechanisms underlying cancer (Kwon et al., 2019). For example, proteomic analyses reveal bacterial influences on immune responses and epithelial integrity, facilitating HPV persistence and cancer progression (Kwon et al., 2019). Together, these omics technologies provide potential biomarkers for early detection, prognosis, and therapeutic targets for cervical cancer (Usyk et al., 2020; Sharifian et al., 2023).

## Conclusion

Vaginal microbiota plays a crucial role in the acquisition, persistence, and clearance of HPV, influencing infection outcomes (Sharifian et al., 2023). HPV infection alters vaginal microbiota diversity, reducing Lactobacillus and increasing anaerobes like *Gardnerella and Prevotella* (Wang et al., 2023; Chen et al., 2020). This shift can promote HPV persistence, raising cervical cancer risk (Zhai et al., 2021; Shannon et al., 2017). A healthy vaginal microbiota promotes a proper vaginal environment and enhances immunity (Wang et al., 2023). Advances in metagenomics, especially shotgun sequencing, have enabled the exploration of microbial diversity in environments like the vaginal tract (Banerjee et al., 2015). Many cancers are linked to microbes, and metagenomic studies help identify disease-causing pathogens, advancing cancer research. Discovering potential cancer targets will stimulate drug therapy research, benefiting patients with HPV infection and cervical cancer (Banerjee et al., 2015).

## Data Availability

The original contributions presented in the study are included in the article/supplementary material, further inquiries can be directed to the corresponding author.
